# Myanmar migrants living along the Thailand-Myanmar border: Experiences related to pandemic and migration decisions

**DOI:** 10.1016/j.jmh.2024.100259

**Published:** 2024-07-26

**Authors:** Wei-Ti Chen, Chengshi Shiu, Rachel H. Arbing, Khin Moe Myint, Khine Myint Oo, Shu-Sheng Lai, David Tanoko, Sarah Oung, Poy Yamada, Saiyud Moolphate, Thin Nyein Nyein Aung, Myo Nyein Aung

**Affiliations:** a School of Nursing, University of California Los Angeles, Los Angeles, CA, USA; b School of Social Work, National Taiwan University, Taipei, Taiwan; c Department of Women's Studies, Chiang Mai University, Chiangmai, Thailand; d Glocal Action Association, Taipei, Taiwan; e Nursing Program, Santa Monica College, Los Angeles, CA, USA; f Department of Public Health, Chiangmai Rajabhat University, Chiangmai, Thailand; g Faculty of Medicine, Chiang Mai University, Thailand; h Faculty of International Liberal Arts and Department of Global Health Research, Graduate School of Medicine, Juntendo University, Tokyo, Japan

**Keywords:** Myanmar, Migrants, Thailand-Myanmar Border, Pandemic, Migration decision, Teenage marriage and pregnancy

## Abstract

**Importance:**

In Myanmar, amid political and civil unrest, droves of Burmese are displaced to neighboring countries including Thailand. Since the COVID-19 pandemic, little is known about the available healthcare services and health and well-being among refugees and migrant workers within resettlement areas along the Myanmar-Thailand border.

**Objective:**

To explore the unmet needs of migrants along the Thailand-Myanmar border during the COVID-19 pandemic and their reasons for leaving Myanmar.

**Design:**

A qualitative study that used focus groups with migrant schoolteachers and school masters was undertaken. An interpretative analysis approach was used to analyze the data from the focus group sessions. The study followed the COREQ (COnsolidated criteria for REporting Qualitative) checklist.

**Setting:**

In July 2022, community stakeholders from migrant schools located in the vicinity of Mae Sot, Thailand were referred to the study team.

**Participants:**

A purposive sample of 17 adult participants was recruited from 4 migrant schools. The participants were schoolteachers and schoolmasters who had traveled from Myanmar to Thailand 1 to 20 years ago.

**Main Outcome(s) and Measure(s):**

Thematic analysis was used to scrutinize qualitative data for the outcomes of health and well-being, barriers, and reasons for migration.

**Results:**

Three main themes were identified: “issues related to the pandemic”, “teenage marriage and pregnancies” and "migration decisions". The issues related to the pandemic included behavior changes in children, a diminished quality of education, and barriers to receiving COVID-19 vaccines and accessing other health care. There were more dropouts due to teenage pregnancy/marriage during the shelter in place mandate. Migration decisions were affected by concerns over health, civil unrest, and military harassment.

**Conclusions and Relevance:**

This study presented the difficulties experienced by Myanmar migrants currently living along the Thailand-Myanmar border. The reasons for leaving Myanmar included health and safety. Suspending education during the pandemic caused more school dropouts due to teenage pregnancy/marriage. Additionally, behavioral changes in children, a diminished quality of education, barriers to receiving COVID-19 vaccines and access to other health care services were reported. Future studies should focus on how migration stress and access to mental health care impact the migrant population.

## Introduction

Myanmar is one of the poorest countries in Southeast Asia. In 2022, Myanmar had a GDP per capita equivalent to 10.65% of the world average (The World Bank, 2017). Nearly 20% of its population lives below the poverty line (The World Bank, 2017). Worldwide, Myanmar is among the countries that spend the least GDP on healthcare, resulting in a severely inadequate healthcare infrastructure, a shortage of health services, and a life expectancy of 66.6 years, The World Health Organization (WHO) (2013) compared to the world average of 72 years. During the COVID-19 pandemic, experts have estimated that more than half of the Myanmar population may be infected with COVID-19 (Nicols, 2021; The Editors of The Japan Time 2021; Chongkittavorn, 2021).

On February 1, 2021, the military in Myanmar initiated a coup against the elected government. In protest, healthcare providers across the nation undertook the Civil Disobedience Movement (CDM) campaign, urging citizens to practice “no recognition” (of the military regime) and “no participation” (in activities related to the military). With the military's refusal to resolve the coup crisis, Myanmar citizens commenced indefinite nationwide general strikes that disrupted functions across social sectors, including healthcare. Political turmoil has become violent and continues to escalate. As published in *The Lancet* (April 7, 2021), a difficult political context has rendered healthcare services inaccessible to patients with chronic illnesses, including HIV (Aung et al., 2021):

- Essential HIV services are reduced or suspended
- Medical supplies are in short supply
- Healthcare facilities are closed or relocated
- Healthcare workers are harassed, jailed, injured, or even killed
- Public transportation is unavailable
- Personal safety is threatened

Interruptions in nearly all domains of Myanmar society, Coup (2021), Tharoor (2021), Han et al. (2021) have led to a “collapse of laboratory testing” (Reuters Staff, 2021) and inaccessible treatments. As a result of violence, another wave of forced migration and displacement is underway.

The latest report of the United Nations High Commissioners for Refugees estimates that a total of 1805,000 individuals have been forced to leave, with 1477,000 being internally displaced and 1086,000 seeking refuge in nearby countries (UNHCR, 2021). Forced migration has long been a serious issue in Myanmar. As an ethnically diverse country, the tensions among ethnic groups due to territorial disputes have resulted in armed conflicts and mass displacements, particularly under the recent military regime. As Thailand shares more than 1500 miles of border with Myanmar, Thailand has been a natural destination for many refugees. Before the coup, an estimated 90,000 refugees lived among nine designated camps at the Thailand border. Since Thailand's government policies severely limited the work opportunities and travel freedoms of refugees in camps, a substantial number of asylum seekers reside outside the camps resulting in equally large populations inside and out.

Wars and conflicts result in tremendous losses among those impacted, and health and human services in resettlements often stumble to meet the needs of refugees. Estimates reflect 31% of refugees have post-traumatic stress disorder (PTSD), 32% have depression, 11% have anxiety, Blackmore et al. (2020) and over 20% experience other or combined mental illness. Additionally, 17%−36% of refugees in resettlements have patterns of substance abuse (Horyniak et al., 2016). Elevated levels of mental health conditions can largely be linked to the violence refugees experienced prior to their forced migration (Scoglio and Salhi, 2021). Together with the loss of social support due to forced relocation, and a lack of social integration in resettlements, untreated mental health conditions may drive the deterioration of the well-being and quality of life of refugees (van der Boor et al., 2020; Posselt et al., 2019).

During the COVID-19 pandemic, refugees were particularly vulnerable (Hayward et al., 2021). In many cases, the pandemic amplified existing structural inequalities resulting in fewer resources available to protect refugees (Im and George, 2022; Matlin et al., 2021; Saifee et al., 2021). Since many people live in poverty, relying on social services to meet daily needs and fulfill key social functions, lockdown measures in resettlement areas further exacerbate economic hardship and social isolation, Hossain et al. (2021), Brickhill-Atkinson and Hauck (2021) and threaten refugees’ mental health and well-being (McGuire et al., 2021). Despite refugees’ heightened vulnerabilities, there is a paucity of empirical studies documenting how forced migration has intersected with the pandemic and created increasingly difficult conditions in which disease prevention competes with personal safety from violence in refugees, especially in resource-limited contexts.

To date, most studies have been conducted in resettlements in high-income countries with established healthcare infrastructures (Bunn et al., 2022; Donnelly et al., 2023; Shad et al., 2023). Little is known about the available healthcare services and health and well-being among migrants within resettlement areas of a developing nation, preventing researchers from developing evidence-based services for refugees from resource-limited countries. In this paper, we aimed to explore the unmet needs of migrants along the Thailand-Myanmar border during the COVID-19 pandemic and their reasons for leaving Myanmar.

## Theoretical framework

Schools have served as a means of integration into host countries for migrant families (Granata et al., 2016). Teachers act as liaisons between families and communities as well as host societies in various ways, including through interpersonal, structural, and cultural aspects. Interpersonally, teachers are trusted to educate children while their parents work, making them key resources for their children's families. Structurally, teachers serve as encyclopedias for the host nation, providing information on its basic infrastructure and guidance on where migrant families can seek help. Culturally, teachers who are knowledgeable about the dual cultural practices of the host country and the migrants’ country of origin educate families on acceptable practices in the host society.

## Methods

### Recruitment and analytic sample

In July 2022, study participants were recruited from Thailand-Myanmar migrant community schools in Mae Sot, Tak Province, Thailand. A purposive sample of migrant schoolteachers and schoolmasters who lived in Mae Sot and its vicinity was used for the study. An onsite collaborator, Glocal Action, assisted the study team in identifying eligible participants who were willing to volunteer for the study.

The study was described, questions were answered, and informed consent was obtained from eligible participants. All participants were told that their involvement in the study would be voluntary and that they would be compensated for their time. The study eligibility criteria were: (a) over 21 years old, (b) resided on the Thailand-Myanmar border, (c) currently serve in migrant schools and (d) able to understand and agree to participate in the study activities. We purposively recruited 17 participants for 5 focus groups (3–4 people per group) from 4 Myanmar community schools in Thailand. The ethical review boards of Mae Tao Clinic (Mae Sot, Thailand) and the University of California Los Angeles approved this study.

### Data collection

Focus groups were conducted in Burmese and were audio-recorded. Each session was conducted in a private room and took between 60 and 90 min to complete. All audio recordings were transcribed to Burmese and representative quotations were later translated to English for publication. To enhance the credibility of the focus group sessions, the study questions were first piloted. The following prompts began each focus group: “Please share with me why you decided to leave Myanmar”, “How did you come here?”, “When and how did you decide to stay here?”, “What are the most concerning health issues in children? How about their parents?”, “How long did you stay here?”, “How did you protect yourself and your family during the COVID-19 pandemic?”

### Data analysis

Inductive interpretative analysis of the focus group data was conducted using Atlas.ti (Scientific Software Development Version 8.0, 2019). The data were coded to identify concept groupings and code trees were used to relate health and migration processes (Spiers et al., 2016; Skinta et al., 2014; Hsieh and Shannon, 2005). Researchers individually reviewed the transcriptions and assigned codes from the code list based on the study themes. To ensure coding reliability, two transcripts were randomly selected for comparison. The research team resolved uncertainties or discrepancies, serving to enhance confirmability of the analysis. Descriptive quotations pertaining to health and migration were selected from the transcriptions. To ensure accuracy, external experts, who were familiar with the culture of Myanmar and migrant health along the Thailand-Myanmar border, reviewed the process of data gathering, thematizing, and study findings. Data saturation was confirmed by the replication of information obtained from participants (Guest et al., 2006). By the 15th to 16th interview, the research staff started noticing that the themes related to pandemic barriers, teenage marriage and pregnancy, and migration decisions were consistently emerging and no new themes were being identified. After a couple more interviews, the research staff concluded that additional interviews were unlikely to provide new insights, indicating that saturation point had been reached.

## Results

The participants’ ages ranged from 21 to 56 years, with an average age of 32 years (SD = 9.72). Eleven respondents were male (64.7%), and six were female (35.3%). Fourteen (82.4%) were teachers in migrant schools, and 3 were principals of schools serving migrant children. Participants had come from Myanmar to Thailand 1 to 20 years ago. Three main themes were revealed: "issues related to the pandemic", “teenage pregnancy and marriage” and "migration decisions". Each theme and its related categories are described below.

### Issues related to the pandemic

#### Behavior changes

Several migrant teachers reported that children experienced behavioral changes due to the lockdown. As all in-person activities were stopped by the Thai government from 2020 to the summer of 2022, children stayed at home with grandparents and/or worked in the fields with parents.

One 32-year-old female teacher shared, “*Some of the children become rude and imitate the behaviors of the people outside the school*.” Another 34-year-old female teacher believed that, “*the children can't concentrate well as they are away from school of so long. Parents do not take care of the kids except let them play cellphone games. We have to catch up so many things. Some students become rude, but all are healthy, as they did not go outside but playing cellphone games.*” Therefore, to aid concentration at school, teachers asked students to meditate twice each day. In large schools with more students, teachers told students to close their eyes and meditate, finding that students immediately quieted as the space was limited for spreading out. Additionally, during school the children sang Myanmar's national anthem. A 28-year-old female teacher shared, “*every morning, we give salute, pray, meditate and talk about life lessons for 10 min*”. During focus group sessions, teachers talked about the importance of education, health, Buddhism, and respect for parents, among other things.

#### Diminished quality of education during the pandemic

Migrant schoolteachers recalled that during the beginning of the pandemic, people in Mae Sot were afraid of COVID-19. However, teachers were willing to travel to students’ houses to provide one-on-one teaching, with parents’ permission. One 48-year-old male teacher stated, “*During the COVID situation, the villagers were very scared. Parents restricted home-based teaching. Village masters did not allow teachers to go outside the school*”. Therefore, teachers visited their students’ houses only to drop off and collect materials for homework. One 34-year-old male teacher shared, “*As we could not teach them due to limitation, we gave the younger kids (things) to draw and do math, gave the materials to the older kids to do math at home. Parents can help too. We could not give too much homework. Just a few things*.” Many parents were uneducated; older siblings who went to school helped with homework.

#### Getting the COVID-19 vaccine and other healthcare

A local teacher group called “Migrant Education Coordination Cancer” contacted the Thai government about COVID-19 vaccine needs in school communities. One 26-year-old female teacher said, “*People are referred to the government facilities to get vaccination because downtown Mae Sot is far away. There is another facility around 42* *km (26 miles) away which is closer where children can get vaccine if the government facilities were crowded*.” However, there was little support after the vaccine was obtained.

### Teenage marriage and pregnancy

The increase in teenage marriage and pregnancy among students was thought to be a result of the pandemic. One 53-year-old female teacher said, “*Most of the kids got married on their own and parents cannot say no but let them marry as parents might also got married in young age. These children are rarely 18 years old. They usually stay with parents and work*.”

Due to the financial hardships experienced during the pandemic, parents let their children work, creating opportunities for these children to meet their teenage partners. Some met their partners via social media. Girls who married during the pandemic were mostly 13 to 16 years old. Several teenage students also gave birth during the pandemic. After they married, the students did not return to school, as many of them had children. Since parents from Myanmar living in Thailand bore the financial burden of supporting newlywed couples, married teens were expected to earn income. As one 56-year-old male teacher shared, “*First, parents cannot control their children. Second, when the school closed, children must work, and they meet at working places and get married. Another thing is mobile phone. All students use social media and start dating. Parents cannot take care of the children due to lack of parents’ knowledge and education*.” In addition, among migrant parent circles, many believed that completion up to grades 4 to 6 was adequate to read and write.

Several senior teachers shared that both they and the parents strongly discouraged migrant teenagers from marrying Thai people because “they are not the same”. Moreover, since migrants in Thailand lack official documents, marriages are ceremonies witnessed by respected elderly people among Burmese tribes. “Usually, they admonish, do the talking and make a wedding arrangement for those who are married”, described a 56-year-old male teacher.

### Migration decisions

#### Health

Families decided to leave their home country for various reasons. Some came to Thailand due to family health issues, while others came to Thailand due to instability within Myanmar. One 36-year-old female teacher shared, “*Families came to Mae Sot and seek healthcare from Mae Tao Clinic when children were sick. One woman knew when she was pregnant with second child while she was treating for TB. She went to Thai hospital and got tested and then found out that she was HIV positive*”. Another 36-year-old male teacher stated, “*People only live here one to two months as they are receiving treatment here. However, it is now hard to connect back to Burma due to the current situation (coup). Especially it is hard to get medicine in Burma beside barriers such as transportation*.”

#### Civil unrest

Many recent migrants from Myanmar came to the Thailand-Myanmar border due to civil unrest. One 27-year-old male teacher said, “*I lived in Bago and left 7 months ago. I do not like the military, so I quit my position as a formal teacher in a government school. As the pandemic is subsided, all schools are open, but there are no experienced teachers, as 95% of the teachers are in civil disobedience movement to support the Aung San Suu Kyi government*”. In addition, many new families with children recently relocated from Myanmar due to the pandemic and the coup. One 34-year-old female teacher shared, “*there were 21 new students from Myanmar this month. Parents lost jobs due to COVID-19 and coup in Myanmar. Also, there is one student came from internal displacement people refugee camp*”.

Other teachers came because they are not able to continue their education in Myanmar. A 21-year-old male teacher shared, “*I am in a normal university and trained to be a teacher; however, because of coup, I cannot continue my college education. I have a friend who is in Mae Sot and encourage me to come. But the situation is not as what I expect in Mae Sot, but I feel bad for these children and their parents, so that is why I stay*”.

#### Military harassment

As younger generations were protesting against the military government, many left due to potential dangers. A 23-year-old male teacher who was Rohingya stated, “*I chose to come to Thailand. I join the coup protest groups and some girls got arrested. The members of the groups were tracked down. Therefore, I need to escape. And with connection, I came here. I can't go to Bangladesh, as it is even worse than here (in Thailand)*. Another 21-year-old female teacher shared, “*I had to take two buses then had to take a motorcycle ride. After that, I have to walk. I did not have my passport, so I had to stay at a place before the timing is right to cross the border*”. She continued, “*there were many check points along the way, and I need to obtain a monastery voucher to pass through the check points. It took about one month to come here. I did not come through Myawaddy as the situation was rough at that time. I came through Karen state. It took one to two days from there to Thailand. I stayed at a refugee camp at a monastery, then I came here.*”

Another 29-year-old, female, Rohingya teacher who came to Mae Sot five years before echoed, “*It was difficult to work in Myanmar. We move because Arakhan state had a conflict with Bangladesh at that time. There are also issues on Rohingya crisis in that region. Therefore, my mother, brother and sister and children are all here. My elder brother is a headmaster in this school. My father passed away, and my husband is also in Mae Sot.*” A 25-year-old male teacher shared, “*I wanted to study in a foreign university for my master. Back in 2019 there was a conflict between Rakhines and Muslims. After the military intervened, the case became complicated. Many families and students were from Rakhine state, they had to run for safety. I saw miliary shot guns and bomb the region, therefore, here, students who came from Rakhine do not have their parents with them but only relatives. I felt from my students, so I stay till now*”.

## Discussion

The Myanmar civil war is ongoing and the number of displaced people is increasing (Chen et al., 2023). Although many migrant health studies have focused on forced migration in other parts of the world, Mohammed et al. (2023), Tusat et al. (2023), Cuadrado et al. (2023), Kurt et al. (2021) there are few recent studies of Myanmar migrants to Thailand (Konig et al., 2022; Sim et al., 2023). While there are some studies on migrant health coverage in Thailand, we found that research on migrant subgroups, such as victims of trafficking and migrant children, as well as on health domains, non-communicable diseases and occupational and mental health is neglected. Taken from the perspective of schoolteachers and school masters, this paper highlights the current situation along the Thailand-Myanmar border which includes the difficulties faced by Myanmar migrants living along the Thailand-Myanmar border, potential reasons for leaving Myanmar, the impact of the suspension of education during the pandemic, and barriers to accessing COVID-19 vaccines and accessing other healthcare services.

During the pandemic, teenagers spent more time on social media and many started dating and married during school closures, fueling student dropout rates. In fact, these “marriages” are not registered and do not appear in any governmental records. However, these young couples are still learning to live independently, and many of them separate within a short time. Sadly, in this conservative population, sexual health is not discussed in the context of formal education (Lwin et al., 2022; Asnong et al., 2018). Many teachers agreed that sexual education should not be mentioned until the Burmese marry. Additionally, their limited proficiency in the local language affects their ability to learn updated essential health knowledge (Davidson et al., 2024).

Due to lockdowns, teachers observed that students no longer behaved and that some started acting out. Meditation was found to be an efficient method for keeping children concentrated on schoolwork. As a common cultural practice in Myanmar, the use of meditation at school worked to teach future generations of Burmese its importance. Additionally, the study participants believed that holding on to Myanmar culture was an obligation of teachers. Praying, meditating and singing the national anthem were used by teachers to keep Myanmar children rooted in their culture.

There are various reasons for migrating from Myanmar. For nearly 75 years, Myanmar's population has endured devastating and compounding intergenerational trauma due to sustained conflict. (Saemiento, 2024) Political instability and violence, as well as ethnic and religious persecution, play an important role in why people in Myanmar leave the county, such as the Rohingya, who faced systematic discrimination, violence and torment. In 2017, many Rohingya refugees began to flee to Bangladesh because of targeted persecution (Rohingya Crisis (UNICEF,) 2023). In our study, several Rohingya participants decided not to travel to Bangladesh, believing the situation to be worse than that of the Thailand-Myanmar border. Others left Myanmar due to a lack of access to basic services, including healthcare. Some participants in this study left due to access to healthcare and medical treatments, while younger college students or recent graduates left due to anticipated government harassment and economic hardship. Others were shut out of Myanmar due to pandemic travel restrictions as well as natural disasters which were difficult to remediate without governmental assistance. In this study, some participants left the country recently, while others left some time ago and stayed.

### Limitations

Several study limitations deserve mention. First, the transition of migration has not been extensively explored, and future studies should investigate the dangers of migration in greater depth. However, this is one of few studies that focus on a mobile Myanmar population currently residing in a low-income country such as Thailand. Second, many acculturation issues were not discussed and warrant further study. However, these Myanmar teachers and school masters shared that they did not plan to stay in the border town for long, returning to Myanmar once the civil war subsided. Thus, acculturation might not be a priority in this particular migrant population. Third, the study may have been subject to researcher bias. In addition, since translation relied solely on a few bilingual researchers, biases may be reflected in transcriptions. Therefore, training local researchers should take precedence in future studies. Finally, this study recruited only 17 schoolteachers and school masters. Future studies should replicate this research using larger samples of Myanmar migrants.

## Conclusions

This qualitative study provides insights into Myanmar migrants’ experiences during the pandemic and why they decided to migrate. Many migrants planned their journeys after the military coup. For healthcare providers who work with migrants, this specific population is worthy of more attention, as their situation is becoming worse as civil war continues. A focus on managing teenage pregnancy and the protection of children among migrant populations is warranted. Future studies should focus on how migration stress and access to mental health care impact the migrant population. Finally, international humanitarian interventions are urgently needed to ensure that migrants from Myanmar have access to necessary care in border countries.

## Key points

**Question:** Why did Burmese leave Myanmar and how did migrants from Myanmar experience living in border towns of Thailand in the context of the COVID-19 pandemic?

**Findings:** Migration decisions were affected by concerns about health, civil unrest, and military harassment. The issues related to the pandemic included behavioral changes in children, higher school dropout due to teenage pregnancy/marriage, diminished quality of education, and barriers to receiving COVID-19 vaccines and accessing other health care services.

**Meaning:** International humanitarian interventions to ensure that migrants from Myanmar have access to necessary health care and education in border countries are needed.

## Funding/Support

Research supported by 10.13039/100007185UCLA CTSI/SON Intramural fund May 2022, NIH/FIC under award number [R01TW012392; PI: Chen, Wei-Ti], NIH/FIC under award number [R21TW011277; PI: Chen, Wei-Ti] and under Award Numbers NIMH [P30MH058107; PI: Shoptaw, Steven J.] Taiwan National Science and Technology Council (111-2410-H-002-126-MY2; PI: Shiu, Chengshi).

## Role of the funder/sponsor

The contents of this article are solely the views of the authors and do not represent the official views of the National Institutes of Health, Taiwan National Science and Technology Council and UCLA.

## CRediT authorship contribution statement

**Wei-Ti Chen:** Writing – review & editing, Writing – original draft, Visualization, Validation, Supervision, Resources, Project administration, Methodology, Investigation, Funding acquisition, Formal analysis, Data curation, Conceptualization. **Chengshi Shiu:** Validation, Supervision, Project administration, Methodology, Investigation, Data curation, Conceptualization. **Rachel H. Arbing:** Writing – review & editing, Visualization, Validation, Resources. **Khin Moe Myint:** Visualization, Validation, Investigation, Formal analysis, Data curation. **Khine Myint Oo:** Validation, Investigation, Formal analysis. **Shu-Sheng Lai:** Resources, Data curation. **David Tanoko:** Validation, Data curation. **Sarah Oung:** Validation, Formal analysis. **Poy Yamada:** Validation, Formal analysis. **Saiyud Moolphate:** Visualization, Data curation. **Thin Nyein Nyein Aung:** Validation, Data curation. **Myo Nyein Aung:** Visualization, Validation.

## Declaration of competing interest

None reported.
